# Understanding the mechanisms underlying obesity in remodeling the breast tumor immune microenvironment: from the perspective of inflammation

**DOI:** 10.20892/j.issn.2095-3941.2022.0547

**Published:** 2023-03-08

**Authors:** Hengjun Zhang, Mozhi Wang, Yingying Xu

**Affiliations:** Department of Breast Surgery, The First Hospital of China Medical University, Shenyang 110001, China

**Keywords:** Breast cancer, obesity, inflammatory mediator, tumor microenvironment, metabolism

## Abstract

Obesity is a well-known modifiable risk factor for breast cancer and is considered a poor prognostic factor in pre- and post-menopausal women. While the systemic effects of obesity have been extensively studied, less is known about the mechanisms underlying obesity-associated cancer risk and the local consequences of obesity. Thus, obesity-induced inflammation has become the focus of research interest. Biologically, the development of cancer involves a complex interaction with numerous components. As the tumor immune microenvironment changes due to obesity-triggered inflammation, an increase in infiltration occurs for proinflammatory cytokines and adipokines, as well as adipocytes, immune cells, and tumor cells in the expanded adipose tissue. Complicated cellular-molecular crosstalk networks change critical pathways, mediate metabolic and immune function reprogramming, and have a significant role in tumor metastasis, proliferation, resistance, angiogenesis, and tumorigenesis. This review summarizes recent research findings on how inflammatory mediators in the *in situ* tumor microenvironment regulate the occurrence and development of breast cancer in the context of obesity. We analyzed the heterogeneity and potential mechanisms of the breast cancer immune microenvironment from the perspective of inflammation to provide a reference for the clinical transformation of precision targeted cancer therapy.

## Introduction

Cancer accounts for 21% of all deaths, making it a major public health problem worldwide. Breast cancer (BC) is the most common malignancy, the incidence of which is increasing. Specifically, BC comprised approximately one-third of newly-diagnosed tumors among women in 2022^1,2^. BC, which is characterized by excessive epithelial cell proliferation, is a highly heterogeneous and multifactorial disease with a genetic predisposition, associated metabolic disorders, and known environmental factors^3,4^. It is commonly acknowledged that the tumor microenvironment (TME) is critically involved in cancer initiation, development, and response to therapies^5,6^.

The TME is a heterogeneous and dynamic cellular milieu composed of resident and infiltrating cells and molecules^7,8^. Collective evidence has revealed the importance of cellular and molecular interaction networks within the TME for cancer cell survival^9^, and recent studies have focused on elucidating the mechanisms by which stromal inflammatory mediators secreted by TME components influence tumor progression.

**Figure 1:**
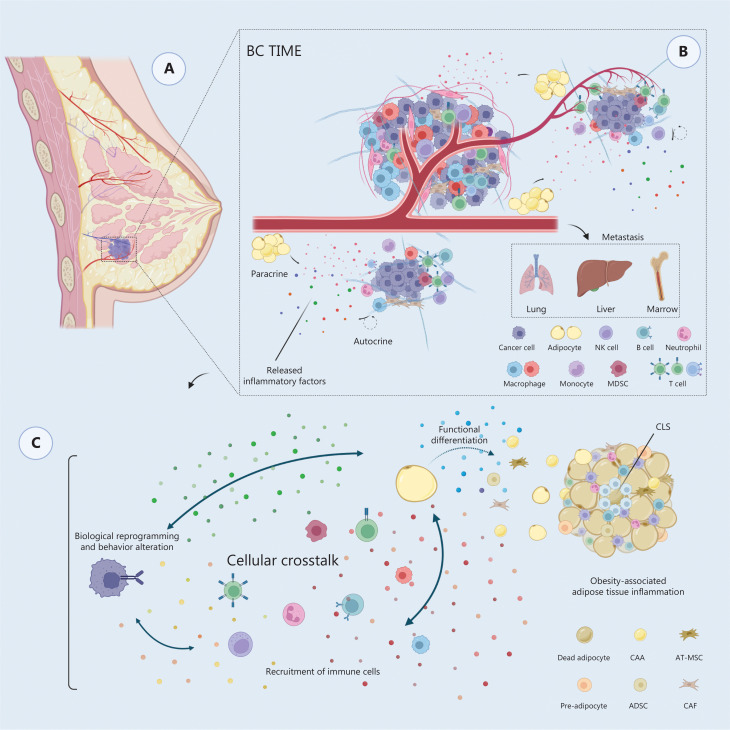
Components and interactions of the BC TME containing adipocytes. (A) The mammary gland is mainly composed of acini and lobules. In the breast, epithelial cells infiltrate around adipose tissue, a unique cell group that maintains the breast morphology. (B) Composition of the TME, including both the physical and biochemical components in the stroma. (C) Schematic representation of interactions between the obesity-associated inflammatory microenvironment and the BC TME. Cellular crosstalk between cancer cells and stromal cell populations leads to various pathologic hallmarks of BC. TIME, tumor immune microenvironment; NK cell, natural killer cell; MDSC, myeloid-derived suppressor cell; CLS, crown-like structure; CAA, cancer-associated adipocyte; AT-MSC, adipose tissue-derived mesenchymal stem cell; ADSC, adipose-derived stem cell; CAF, cancer-associated fibroblast.

As shown in **Figure 1**, the biological properties of tumor cells are inextricably regulated by dynamic shifts in systemic metabolic status due to intimate interactions between tumor cells and their surroundings. Obesity is a chronic metabolic disease that is defined by the accumulation and reprogramming of adipose tissue. Extensive studies have reported a correlation between obesity and breast tumor-related events, such as poor survival, therapy resistance, recurrence, and metastasis^10–19^; however, the underlying mechanisms have not been established. Notably, obese patients usually have impaired inflammatory responses and immune regulation. Moreover, a correlation exists between body mass index (BMI) and the proinflammatory state of breast tissue (*P* = 0.004)^20^, which has a significant role in worsening the prognosis^21–23^. Inflammation is one of the hallmarks of carcinogenesis, and researchers have revealed that an inflammatory milieu could favor BC initiation and invasiveness^24,25^. Obesity stimulates adipose tissue and BC cells (BCCs) to release inflammatory mediators, predisposing the BCCs to a proinflammatory state, driving oxidative stress, and creating paracrine and autocrine feedback loops^26^. In summary, the obese environment creates a distinctive inflammatory milieu in adipose tissue that favors BC initiation, primary growth, invasion, and metastatic progression in conjunction with systemic endocrine alterations.

Tumor progression is typically orchestrated by crosstalk between the tumor and stroma^27^. The TME in patients with BC is dominated by stromal cells, which actively participate in tumorigenic malignant transformation^5,12^. Breast ductal epithelial cells are immersed in an adipose environment containing plentiful, specialized adipocytes^29,30^. The intimate bidirectional relationship between BCCs and para-cancerous adipocytes has been verified^31,32^. In addition to being an energy resource, adipose tissue is a bioactive organ associated with endocrine, metabolic, and immune systems that regulate systemic energy and metabolic homeostasis, as well as contribute to the composition of the extracellular matrix (ECM) through a complex signaling network^33,34^. Adipocytes secrete numerous soluble inflammatory mediators that bind to cancer cell receptors and affect BCCs via paracrine signaling pathways^35,36^. Preclinical models have linked these secreted factors in primary BC to reduced differentiation and poor clinical outcomes^37,38^. Moreover, adipose tissue is composed of heterogeneous cells, such as adipose-derived stem cells (ADSCs), cancer-associated adipocytes (CAAs), and tumor-associated macrophages (TAMs), that are reprogrammed to exhibit different molecular and cellular characteristics during BC initiation and progression, and increase cancer cell proliferation and invasion capacities by producing inflammatory mediators^39,40^. Defining these unique differences in adipose components might contribute to a better understanding of how the immunosuppressive BC TME arises.

The mechanisms underlying obesity and inflammation influencing BC are largely unknown. Adipose tissue is an essential modulator of BC biology. Understanding how the obesity-associated *in situ* breast inflammatory microenvironment controls cancer cell behavior and identifying the regulatory elements involved could aid in developing prognostic or therapeutic targets.

## Tumor stemness and carcinogenesis in the obese inflammatory microenvironment

**Figure 2:**
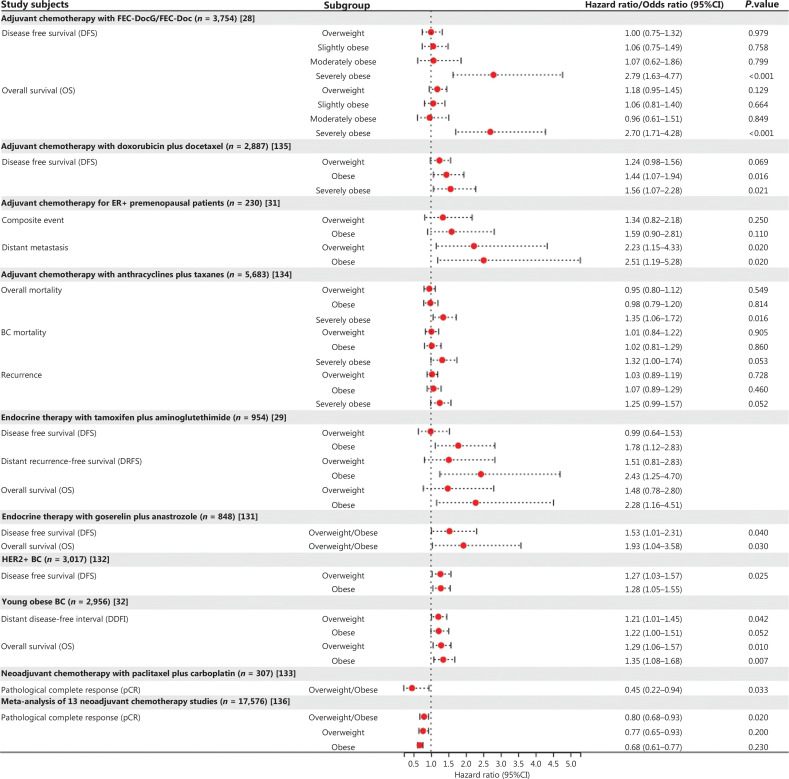
Forest plot of hazard or odds ratio for clinical characteristics in each subgroup from different studies. Each subgroup used the underweight/normal weight group as a reference. FEC-DocG, 5-fluorouracil, epirubicin, cyclophosphamide - docetaxel, gemcitabine; FEC-Doc, 5-fluorouracil, epirubicin, cyclophosphamide - docetaxel; CI, confidence interval. According to the WHO criteria, patients are classified as underweight/normal weight (BMI < 25 kg/m^2^), overweight (BMI = 25–29.9 kg/m^2^), slightly obese (BMI = 30–34.9 kg/m^2^), moderately obese (BMI = 35.0–39.9 kg/m^2^), and severely obese (BMI ≥ 40.0 kg/m^2^).

Obesity increases the BC incidence, particularly for post-menopausal women^41,42^ (**Figure 2**). The risk of breast tumor malignant progression may be accelerated by alterations in the mammary tissue prior to tumor formation^40,43^. BC emerges due to complicated interactions between the environment and genetics that modify the immune and inflammatory systems to promote carcinogenesis.

### Tumorigenesis

Inflammation of white adipose tissue (WAT) is correlated with higher estrogen and aromatase gene expression, both of which are significant risk factors for BC in obese postmenopausal women. Martínez-Chacón et al.^38^ reported that IL-10 inhibits aromatase gene expression in mesenchymal stem cells (MSCs) and ADSCs by suppressing TNF-α-stimulated ERK1/2 activation. Increased adiposity caused by ovarian hormone deficiency in ovariectomized mice results in enhanced expression of aromatase in mammary adipose tissue mediated by decreased local IL-10. Similarly, Alhallak et al.^44^ reported that compared to normal weight women, IL-10 and FOXP3+ T regulatory levels in breast adipose tissue of obese premenopausal women were upregulated along with the expression of tumor suppressive markers in breast tissue. *In vitro* application of IL-10 was shown to promote apoptosis and inhibit proliferation in the mammary epithelial cell lines, MCF10A and HMEC. In summary, IL-10 may act as a potential protective mediator against BC; however, the current evidence is insufficient and additional research is warranted. Furthermore, the above-cited studies revealed a difference in obesity-associated oncogenesis between pre-menopausal and post-menopausal women, which may reflect hormone levels.

Another study showed that CCL2 augments estrogen production and cancer risk by activating the aromatase promoter, pI.4, via the ERK1/2 pathway. The mammary fat pads of animals with weight gain have significantly higher amounts of IL-6, CCL2, and leptin, which are positively linked with mammary aromatase reporter activity in experiments with human primary breast adipose stem cells (ASCs) and mouse mammary adipose explants^45^. Additionally, local estrogen biosynthesis via aromatase and inflammation are linked to COX-2-dependent PGE2 production in obese breast tissue. Gonçalves et al.^46^ reported that an obesogenic diet augments proinflammatory mediators (CCL2, IL-6, COX-2, and PGE2) in breast tissue, along with the formation of crown-like structures of the breast (CLS-B) and upregulation of local aromatase and estrogen, which accelerate COX-2-dependent breast tumor formation.

Other inflammatory mediators associated with obesity that may contribute to an increased risk of BC are also of concern. Human triple-negative BC (TNBC)-derived conditioned media increases the expression of the CAA-related cytokines (CCL2, CCL5, IL-1β, and IL-6), as well as the immune inflammatory regulators, COX-2, HIF-1α, VEGF-α, and PD-L1 in ADSCs during adiposity. The epithelial-mesenchymal transition (EMT) biomarker, Snail, and activation of Smad2 and NF-κB are also correlated with the CAA phenotype, which in turn has a role in tumorigenesis^47^. Tumor-bearing breast adipose tissue has a distinct physiologic state compared with tumor-free breasts, and the capacity to secrete more IL-8, differentially express genes associated with inflammation and the integrin and Wnt signaling pathways, thereby contributing to tumorigenesis^27^. Roubert et al.^48^ showed that BMI is positively correlated with the release of IL-1, IL-6, and TNF-α, which stimulates Wnt signaling in mammary tissue and promotes BC^48^. Furthermore, reduced breast epithelial tumor suppressor TGF-β1 activity and elevated TGF-β1 within the ECM of obese mammary tissue *in vivo* enhance BC risk, possibly due to interactions with decorin expressed on macrophages surrounding the ducts and lobules in obese breast tissue^49^.

### Tumor cell stemness

Some stem or progenitor cell types may increase the probability of developing BC^50^. One study demonstrated that visfatin induces monocyte M2 differentiation via ERK/CXCL1 and enhances BCC tumorsphere formation and stemness^51^. In addition, when co-culturing human-derived adipocytes with established and primary BCCs, the adipocytes secrete more IL-6 to promote Src activation, thus leading to upregulation of Sox2, c-Myc, and Nanog, which results in the emergence of tumor stem cell traits^52^. As demonstrated by Tiwari et al.^53^, obesity induces a pro-inflammatory metabolic activation phenotype in mammary adipose tissue macrophages *in vivo*, leading to the secretion of IL-6 in an NADPH oxidase 2-dependent manner and promotes stem-like features to alter the niche and support tumor formation via glycoprotein 130 signaling in TNBC cells. ADSCs exhibit tropisms similar to MCF-7 cells, in which secreted macrophage inflammatory protein (MIP)-1δ and MIP-3α induce tumorsphere formation *in vitro* and promote tumorigenicity *in vivo*^54^. In addition, adipokines may have an important role in forming stem characteristics. Avtanski et al.^55^ showed that resistin induces MCF-7 and MDA-MB-231 cells to acquire cancer stem cell (CSC)-like properties. The adipokine, adipsin, promotes proliferation and CSC-like characteristics in xenograft models, suggesting that adipsin contributes to the TME and CSC niche in breast malignancies^56^. A-FABP released from adipose tissue promotes tumor stemness by activating the IL-6/STAT3/ALDH1 pathway^57^. Mishra et al.^58^ also showed that leptin promotes BCC stemness, probably through the induction of TGF-β1 expression and secretion.

## Inflammatory mediators from expanded adipose tissue affect BC growth and survival

In addition to the roles in immune regulation and inflammatory responses, obesity-derived inflammatory mediators also facilitate BCC growth and survival. In fact, adipokines may be involved in mediating this effect. Leptin stimulates the proliferation and viability of both ER-positive and TNBC cells, while also promoting anti-proliferative, cytotoxic, and pro-apoptotic effects in MCF-7 cells, similar to TNF-α and IFN-γ^59^. Accordingly, inflammatory mediators may affect diverse molecular subtypes of BC via different mechanisms. There is also evidence that leptin treatment of MCF10A cells leads to increased survival and reduces apoptosis associated with FAK/AKT phosphorylation^60^. Visfatin is an adipokine, the serum levels of which are elevated in various cancers^61^. NAD generated by extracellular visfatin increases SIRT1 activity and p53 deacetylation, which induces BCC proliferation^62^. Visfatin-mediated proliferation of MCF-7 and MDA-MB-231 cells is related to the ERK1/2 and AKT signaling pathways^63^. Additionally, type I IFNs promote aromatase synthesis in adipose tissue surrounding BC and recruit the HIF1α-IFI16/204-PRMT2 complex to the aromatase promoter, PI.3/PII, which amplifies E2-dependent BC proliferation^64^.

Chemokines maintain an ability to induce targeted chemotaxis in effector cells, and the role of chemokines in promoting tumor proliferation has gained increased attention. In a xenograft model, CCL2-induced inflammation dramatically increases tumor development and facilitates the establishment of a desmoplastic stroma in a xenograft model by early recruitment of macrophages and CAFs into the TME. Fibrocytes may be a potential target inside the TME to minimize tumor fibrosis and improve the treatment response in obese BC patients^65^. Kim et al.^66^ reported significant IL-6 staining in fibroblast-like cells within the peritumoral region, in addition to a positive correlation with CAF markers, thus implying that IL6-positive fibroblast-like cells may originate from pre-adipocytes and mature adipocytes.

Chemokines mediate tumor resistance by participating in the formation of *in situ* regional fibrosis, which in turn leads to tumor growth. Xenograft-bearing mice fed a high-fat diet (HFD) exhibit increased expression of IL-6 and TNF-α to sustain the local chronic inflammatory microenvironment and favor M1 macrophages, which together with nicotine increase macrophage infiltration and anti-inflammatory cytokines, such as IL-10, IL-13, and IL-4, promote BC growth and invasive behaviors^67^. IL-6 and CXCL1/2/3 are increased and MCP-2 is decreased in human BC-conditioned media, and this effect correlates with tumor stage and histologic grade^68^. The inflammation-related genes, IL6, Ptx3, IL33, and Timp1, are upregulated after 3T3-L1 adipocytes are co-cultured with BCCs, thus promoting proliferation in ER-positive BCCs^69^. Obesity-triggered chemokines are involved in tumor growth; however, there is a lack of in-depth studies with respect to the specific underlying mechanisms.

ADSCs are an important member of the adipose microenvironment. Crosstalk between the secretome and TME may have a role in tumor growth. ADSCs expressing CXCR4 are recruited by SDF-1α (CXCL12) secreted from chemoresidual TNBC cells. These ADSCs then initiate signaling to drive tumor cell proliferation by secreting FGF2 and activating ERK^70^. ADSC-secreted CXCL5 is a key factor in promoting the proliferation of MCF-7 and MDA-MB-231 cells^71^. Another study showed that ADSCs facilitate the growth of MCF-7 and ZR-75-30 cells *in vivo* by secreting the pro-angiogenic cytokines, CXCL1 and IL-8, but have a limited effect on MDA-MB-231 cells^72^. Furthermore, co-injection of c-Kit^+^ ADSCs with 4T1 or endothelial progenitor cells *in vivo* elevate IL-3 and SDF-1 levels and increase tumor volume^73^. These results may provide a foundation for inhibiting ADSCs or the secretome in the clinical setting.

## Obesity-associated inflammation promotes tumor angiogenesis

Angiogenesis is of great significance for tumor development because blood vessels in the TME supply tumor cells with nutrients and oxygen^74^. Tumor cells have been shown to attract bone marrow-derived vascular endothelial progenitor cells (BM-EPCs) and promote angiogenesis via the VEGF/HIF-1α pathway^6^. Pro-inflammatory mediators also maintain angiogenesis and tumor progression, regardless of VEGF blockade^75–77^. Many of these variables are also increased in obese patients, thus creating a complex microenvironment where cytokines, chemokines, and adipokines are released to cause inflammation and recruit additional pro-inflammatory and pro-angiogenesis mediators^78–80^. Numerous studies have reported correlations between obesity and enhanced tumor angiogenesis, highlighting the importance of angiogenesis for the progression of obesity-driven BC^81,82^. To suppress tumor angiogenesis, several anti-angiogenic agents have been developed, which unfortunately have not yielded the desired results in clinical trials^83^.

One of the most pressing issues in the effort to improve therapies is how to overcome resistance to anti-angiogenic drugs in cancer patients. Using a zebrafish model of metastasis, Rodriguez et al.^84^ showed that IL-8 generated by breast adipocytes might synergize with LFA-1, integrin, CCL5, or CCL2 to transform the BC microenvironment into a pro-inflammatory and pro-angiogenic state, while inducing resistance against anti-VEGF treatment in ER-positive BCCs. Incio et al.^85^ reported that *in vivo* IL-6 inhibition and FGF-2 adjustment eliminate obesity-induced resistance to anti-VEGF therapy by impacting tumor cell growth, restoring tumor vasculature, alleviating hypoxia, and diminishing immunosuppression. Hsieh et al.^86^ also proposed that IL-8 improves the pro-angiogenic effects of breast adipocytes.

VEGF-A promotes neovascularization, increases vascular permeability, and is closely related to angiogenesis. The obesity-associated inflammatory cytokines, TNF-α, INF-γ, and leptin, increase VEGF-A levels and affect angiogenesis in MDA-MB-231 cells^59^. Elevated IL-6 secreted by adipocytes co-cultured with cancer cells promotes angiogenesis via upregulation of STAT3 and VEGF^52^. Moreover, c-Kit+ ASCs also improve VEGF levels and vascular formation^73^.

In addition to directly promoting VEGF-A expression, several potential mechanisms may also mediate tumor angiogenesis. Kolb et al.^87^ reported that obesity increases the number of tumor-infiltrating macrophages with activated NLRC4 inflammasomes and boosts IL-1β production, thus promoting angiogenesis and cancer progression by upregulating ANGPTL4 expression via NF-κB and MAPK activation. Indeed, targeting ANGPTL4 separately or in conjunction with anti-VEGF treatment may be a more effective strategy for treating obese patients with BC. Another study showed that a pro-inflammatory setting with elevated IL-6 and IL-12 expression and upregulated stress-induced pp38 MAPK and pERK1/2 encourages BC angiogenesis in HFD xenografts^67^. Moreover, WAT-derived GM-CSF and MMP9 promote immunosuppression and intratumor vascularization^88^.

Inflammatory mediators also promote tumor angiogenesis by affecting the activity and function of vascular endothelial cells. Wang et al.^72^ co-injected human ADSCs and BCCs *in vivo* and demonstrated that ADSCs secrete CXCL1 and IL-8 to increase the migration and tube formation of human umbilical vein endothelial cells (HUVECs) by signaling via the receptors, CXCR1 and CXCR2, which in turn enhance angiogenesis and tumor growth. In addition, paracrine activation of integrin β1-ERK1/2-HIF1α-VEGFA signaling by MSCs injected into adipose tissue promote HUVEC proliferation, migration, and angiogenesis^89^. Obesity results in the release of increased inflammatory mediators acting on vascular endothelial cells to promote viability, thereby favoring angiogenesis.

## Obesity-induced inflammation transforms the mammary gland into a microenvironment conducive to tumor aggressiveness and metastasis

Metastasis remains the predominant cause of the high mortality rate among BC patients, although the 5-year survival rate has greatly increased over the past few decades^1^. Tumor dissemination is a complicated process in which tumor cells escape from the primary site, persist in the peripheral circulation, extravasate to distant sites, and ultimately multiply within the target organs. Several factors, including tumor cell characteristics and the local microenvironment in the target area, may affect metastasis. At present, insights into the underlying mechanisms by which obesity-induced metastasis occurs are largely lacking, yet comprehending the connection between obesity and metastasis is imperative to develop targeted therapies for obese individuals.

### Chemokines

Chemokines and chemokine receptors have key roles in TME formation, leukocyte recruitment, angiogenesis, and metastasis, and also mediate interactions between tumor cells and tumor surroundings^90,91^. Targeting CC-chemokine ligand 5 (CCL5) *in vitro* reverses the enhanced motility of TNBC cells upon co-culturing with human adipocytes. CCL5 is also detectable in TNBC peri-tumoral adipose tissue and correlates with lymph node positivity, distant metastases, and reduced overall survival among patients with TNBC^92^. Adipocytes within breast tissue release increased CCL2 when lipid metabolism is altered. Patients with high expression of atypical chemokine receptor 2 (ACKR2) may bind and internalize CCL2 for intracellular degradation to limit tumor metastasis, thus leading to low recruitment of CCR2+ monocytes along with high recruitment of CCR2+ NK cells^93^. This result may provide a biomarker for the clinical management of patients. The interactions between adipokines and chemokines also has an essential role. Wang et al.^51^ demonstrated that visfatin-induced M2 differentiation in monocytes via CXCL1/ERK phosphorylation enhance BCC viability, migration, and EMT *in vitro* and in orthotopic mouse models. *In vitro* experiments showed that leptin leads to BC bone metastases by stimulating the SDF-1/CXCR4 axis, which has been validated using clinical samples^94^. Moreover, 4T1 cells treated with HFD mouse adipose-conditioned medium exhibit enhanced migration ability through CXCL12 and CCL25, with an overall decrease in immune cell infiltration and activation in tumor sentinel lymph nodes, suggesting a high risk of metastasis and immune escape^95^. In summary, obesity may be a risk factor for chemokines to enhance tumor metastasis and immunity exemptions.

Numerous substances, including chemokines, metabolites, and exosomes, are secreted by CAAs and may contribute to BC malignancy^96^. Human CAA-derived leukemia inhibitory factor (LIF) promotes BCC migration and invasion, elevates the expression of BC-derived CXCL1/2/3/8, and activates ERK1/2 signaling to further drive LIF expression in CAAs via NF-κB/STAT3 transcription. This effect forms a positive feedback loop between adipocytes and BCCs. Targeting LIF in combination with CXCR2 greatly decreases *in vivo* BC lung metastasis^97^. Another study showed that ectopic IL-8 expression in tumor-adjacent adipocytes converted adipocytes into CAAs, with high levels of NF-κB and NF-κB targets (leptin/IL-8/IL-1 and an active pre-oncogenic STAT3-dependent EMT phenotype), on BCCs and in orthotopic tumor xenografts in mice. Additionally, inactivating CAAs and blocking the IL-8-dependent pro-carcinogenic effects was shown to be achieved by targeting CXCR1/2 in mammary adipocytes *in vitro*^98^. CAA-derived molecules may promote tumor progression through communication with chemokines secreted by tumor cells.

### NF-κB signaling pathway

The relationship between the NF-κB signaling pathway and pro-inflammatory mediators has been studied extensively and has been shown to regulate inflammation and cellular immune responses. Microarray data have revealed that co-culture with adipocytes induces migration and pro-inflammatory genes involved in NF-κB signaling in the MDA-MBA-231 cell line^99^. The expression of obesity-related mediators (SphK1 and S1PR1) is enhanced in metastatic lesions of syngeneic and spontaneous breast tumor obese mice, along with elevated levels of TNF-α and IL-6. Targeting the SphK1/S1P/S1PR1 axis mediated by lipid metabolism attenuates key pro-inflammatory cytokines and macrophage infiltration as well as obesity-induced tumor progression^100^. Treatment with 17β-estradiol, leptin, IL-6, and TNF-α (ELIT) for obesity-related inflammation also decrease mitochondrial function and increase oxidative stress, aggressiveness, and motility in cell lines with low estrogen receptor beta (ERβ) expression^101^. Consistent with this finding, human and mouse adipocyte-derived conditioned media increase BCC migration and proliferation by activating the PI3K-AKT-mTOR pathway and upregulating the expression of target genes, such as TNF-α, IL-1, and IL-6, suggesting that tumor-derived molecules regulate the TME in addition to the tumor, thereby aggravating diseases^102^. Another study indicated that upregulation of IL-6 and TNF-α is disrupted by obesity^95^, indicating that obesity may affect the NF-κB signaling pathway through different mechanisms, which warrant further study.

In addition to directly affecting tumor cells, the NF-κB pathway is also involved in adipocyte-mediated tumor progression and metastasis via paracrine signaling. The growth and migration of 4T1 cells are stimulated by 3T3-L1 adipocyte-conditioned medium, which could be rescued by inhibiting macrophage chemoattractant protein (MCP-1/CCL2), IL-6, IL-1, and plasminogen activators^86^. Pre-adipocytes upregulates IL-6 secretion and promotes proliferation, migration, and invasion of MCF10DCIS.com cells, which can be attenuated by blocking IL6-mediated cross-linking *in vitro* and in a xenograft tumor model^66^. These results indicated that human adipocyte-derived IL-6 and leptin boost BC proliferation and metastasis, and induce an EMT phenotype *in vitro* by activating PI3K-AKT and IL-6/JAK-STAT3 signaling^103,104^. Additionally, using a zebrafish model of metastasis, Rodriguez et al.^84^ demonstrated that the release of IL-8 by breast adipocytes might cause neutrophils to adopt a pro-tumorigenic phenotype, which would then promote the dissemination of ER-positive BCCs. In conclusion, the NF-κB signaling pathway may promote tumor metastasis by affecting tumor cells and adipocytes.

### ADSCs

Multipotent ADSCs have attracted extensive attention because of the regenerative properties in plastic surgery; however, these features are also connected to metastasis and tumor initiation, raising concerns about safety in clinical applications^105^. Human ADSC-secreted IL-6 significantly stimulates proliferation, EMT, transmigration, and 3D invasion of human primary normal and tumor epithelial cells^106^. Blocking ADSC/Th2-derived IL-4 depleted metastatic ADSCs and elevated the expression of DUSP4 by inhibiting NF-κB and impairing the RAS/P38-MAPK pathway, thus delaying the proliferative and invasive phenotype of BCCs and driving the transformation of metastatic cells into non-metastatic cells^107^. By cross-reacting with the transforming growth factor (TGF)-β/Smad and PI3K/AKT pathways, the paracrine impact of ADSCs promotes EMT of MCF7 cells^108^. Moreover, Chang et al.^109^ discovered that adipose tissue-derived mesenchymal stem cells (AT-MSCs) obtained from type 2 diabetes mellitus (T2DM) donors differentiate into cancer-associated fibroblasts (CAFs) when co-cultured with BCCs under hypoxic conditions, in turn promoting BCC proliferation and *in vivo* metastasis, as well as the expression of fibroblastic markers, which was associated with TGFβ-Smad2/3 signal activation. Understanding the inflammatory characteristics of ADSCs may help develop applications in the clinical setting.

### Immunomodulatory factors

Immune cells interact with tumor cells via immunomodulatory factors in the TME, which affects tumor behavior and treatment efficiency. A diet-induced obese mouse model showed elevated collagen production and proliferative rates of lung stromal cells, and the upregulated CSF2- and TGF-β1-induced recruitment and invasion of myeloid cells, as well as an immunosuppressive macrophage phenotype, respectively, which are correlated with pre-metastatic niche formation and lung metastasis of primary mammary tumors^110^. As a growth factor for hematopoietic and immune cells, granulocyte-macrophage colony stimulating factor (GM-CSF) mobilizes stem cells and causes macrophage/granulocyte differentiation in addition to regulating inflammation and autoimmune disease^111^. Liu et al.^112^ identified G-CSF and the G-CSF target gene matrix metallopeptidase (MMP) 2 and 9 in CAAs through transcriptomic sequencing. Treatment of TNBC cells with G-CSF enhance EMT, migration, and invasion through activation of STAT3 in cooperation with IL-6 and GM-CSF. TGF-β activates ERK and phosphorylates SMAD4 in obese patients, which allows USP9x to block the binding and mono-ubiquitination of SMAD4, thereby maintaining SMAD4 while promoting TGF-β/SMAD3-mediated transcription of EMT markers. Free fatty acids (FFAs) further facilitate the process and promote TGFβ-dependent cancer invasion and metastasis^113^. In addition, Wolfson et al.^114^ reported that increased TGF-β1 in the mammary adipose tissue of obese mice activate SMAD3 signaling and inhibit miR-140 transcription, thus preventing miR-140 from targeting SMAD3 for degradation, resulting in the formation of a fibrotic microenvironment that impacts stemness, invasion, and proliferation of ductal epithelial cells. According to Quail et al.^115^, obesity-produced IL-5 boosts CSF2 expression by IL5Rα+ monocyte and neutrophil transport to the lungs of mice. In addition, obesity-related pro-metastatic effects can be reversed by blockade of GM-CSF or weight loss. After co-culturing with BC, human-derived WAT-derived ADSCs dramatically upregulate the expression of GM-CSF and MMP9, neutralization of which significantly reduced local and metastatic BC progression *in vivo*. This process is related to reducing macrophages and myeloid-derived suppressor cells (MDSCs) and can be inhibited by metformin, suggesting that proteins may be novel targets for this widely used drug^88^. Among all immune cells present in the BC TME, monocytes or TAMs account for the majority and are correlated with obesity and a poor prognosis in BC patients. Targeting or reversing the differentiation into monocyte-macrophage immunosuppressive phenotypes and the immunoregulatory factor, TGF-β, may have clinical application.

### Adipokines

Adipokines mediate tumor development through tumor-stromal interactions in the TME. As a cytokine-like hormone, leptin serves as a bridge between BC and obesity. Knockdown of ObR in BCCs induce a less proliferative and invasive tumor phenotype^116^. Treatment with leptin induce EMT, migration, and invasion of BCCs and MCF10A cells, which correlate with increased FAK/AKT phosphorylation^60^. Gelsomino et al.^117^ showed that aromatase inhibitor (AI) anastrozole-resistant MCF-7 BCCs (AnaR) express higher levels of leptin along with AnaR receptors and that targeting leptin signaling reduce growth and viability. Another study showed that leptin reduced cell aggregation and increased cell proliferation, migration, invasion, and EMT of cancer and epithelial cell lines, possibly by inducing TGF-β1 expression and secretion^58^. MDSCs are known promoters of cancer progression. Obesity-induced inflammation upregulates leptin *in vivo*, which induces the accumulation of excessive MDSCs and further facilitates spontaneous growth and metastasis by suppressing the activation and function of tumor-reactive T lymphocytes^118^.

Other types of adipokines also have an important role in tumor invasion and metastasis. Resistin stimulates motility in MCF-7 and MDA-MB-231 cells through cytoskeletal remodeling and EMT reprogramming^55^. A-FABP released from adipose tissue directly targets BCCs and enhances tumor aggressiveness *in vivo* by activating the IL-6/STAT3/ALDH1 pathway^57^. In addition, A-FABP expression in CD11b+F4/80+MHCII-Ly6C- phenotype TAMs facilitates protumor IL-6/STAT3 signaling by regulating the NFkB/miR-29b pathway, and A-FABP deficiency significantly decreases the growth and spread of breast tumors in transgenic and syngeneic tumor models^119^. Consequently, adipokines may be a potential target for the precise treatment of BC, but specific mechanisms need to be investigated further.

The impact of obese stroma on tumor cells and exposure to heightened inflammatory mediators present in the TME lead to alterations in the mammary epithelium that possibly predispose women to the developing of more aggressive BC.

## Inflammation triggered by obesity makes the breast TME resistant to therapeutic approaches

A significant barrier to treating cancer is the resistance to chemotherapy and molecular-targeted therapies. Despite high response rates to initial therapy, many cancers eventually lose sensitivity to original treatment methods, resulting in metastasis and mortality. The TME is crucial in intrinsic drug resistance pathways^120^, and patients who are obese are more likely to develop treatment resistance^98,121^. Prior research has demonstrated that peri-tumor adipocytes contribute to the development of a radio-resistant phenotype in BC^122^. A recent study also found adipocytes to actively convert the chemotherapeutic agent daunorubicin into a less effective metabolite^123^.

### Resistance to chemotherapy and endocrine therapy

Tumors adopt various pathways to inhibit chemotherapy efficacy, in which obesity-associated inflammatory pathways may be involved. The anti-proliferative effects of tamoxifen on MCF-7 cells are counteracted by co-culture with mature adipocytes from obese women, which significantly increase expression of TNF-α and IL-6^124^. Further evidence supports the role of obesity-induced inflammatory responses (IL-6, TNF-α, and leptin) in tamoxifen-acquired BC resistance^85^. Human ADSC-secreted CXCL1 *in vitro* downregulates miR-106a and upregulates ABCG2, a transporter of doxorubicin efflux in TNBC, thus conferring doxorubicin resistance^125^. The inflammatory adipocytokines (leptin, CCL2, IL-1β, and resistin) secreted by adipocytes and FFAs released by MDA-MB-231 cells confer acquired doxorubicin resistance in BC by generating inflammation and lipid metabolic reprogramming in the TME^61^. Mentoor et al.^126^ suggested that obesity-induced resistin inhibits *de novo* fatty acid synthesis and lipolysis in mammary adipose tissue of mice, further aggravating local inflammatory reactions via the NF-κB pathway and promoting TNBC survival together with reduced doxorubicin efficacy in a paracrine manner. In addition, the adipokine, visfatin, enhances cell viability and prevents the reduction of survivin, a well-known candidate for chemoresistance of cancer cells, in TNFα-induced apoptosis and PARP cleavage^63^.

### Resistance to radiotherapy

Radiotherapy is a mainstream BC treatment, and the TME is known to influence radiosensitivity. Inflammation is a crucial aspect of radiotherapy because inflammation can be triggered by released debris and the production of proteins induced by radiotherapy to enhance immune system elimination of cancer cells^127^. Autotaxin (ATX) is mainly secreted by mammary adipocytes in BC; however, as a component of the wound healing response, the ATX-lysophosphatidate (LPA)-inflammatory cycle shields cells against radiation-induced death and facilitates fibrosis^128^. Exposure of rat- and human-derived adipose tissue to γ-radiation produces a significant inflammatory response, and inflammatory cytokines secreted by breast tumors, including IL-1β, IL-6, IL-10, and TNF-α, stimulate ATX secretion by activating the NF-κB pathway. This response, in turn, generates LPA and stimulates further activation of NF-κB, which results in a positive feedback loop that promotes BC growth and metastasis^129^. Meng et al.^130^ confirmed that repeated fractions of radiotherapy activate the above-outlined cycle, further increasing concentrations of VEGF, CXCL10, CCL11, and G-CSF in irradiated fat pads, thereby reducing the efficacy of additional fractions. Moreover, targeting ATX in a syngeneic orthotopic mouse model of BC decreased CCL11, IL-9, IL-12p40, M-CSF, and IFN-γ, which reversed pro-survival signals and radio-resistance in cancer cells. Furthermore, ATX inhibition is synergistic with irradiation and doxorubicin to decrease tumor growth and the number of Ki67-positive cells^131^. Inhibition of the adipose-related ATX pathway restores the pro-inflammatory properties of radiotherapy, and is therefore effective when combined with other therapeutic modalities.

### Potential roles of inflammatory mediators in modulating immune responses

In recent years the development of immunotherapies based on immune checkpoint blockade (ICB) has dramatically reshaped the landscape of cancer therapy; however, despite the improved prognosis in a subset of cancer patients using currently-available immunotherapeutics, the response rate among TNBC patients is < 20%^132,133^, largely owing to the immunosuppressive TME. Therefore, a major focus of research involves discovering and addressing variables that contribute to the immunosuppressive TME. Obesity may be one such variable, with evidence suggesting that obesity perturbs the immune system and allows macrophages and T cells to differentiate into phenotypes that favor tumor growth^22^. Nevertheless, one study also showed that obese cancer patients have better overall survival, progression-free survival, and response rates after ICB treatment^134^. Accordingly, further study is needed to determine how obesity affects the outcomes of cancer patients receiving immunotherapy.

T and B cells are the main types of lymphocytes and have various biological functions, such as the direct killing of target cells, the production of cytokines and antibodies, and immune regulation. Gibson et al.^135^ reported that obesity increases the concentration of CXCL1 in the mammary TME, drives CXCR2-mediated chemotaxis, and accumulates granulocytic MDSCs (G-MDSCs) to stimulate CD8+ T-cell apoptosis via Fas/FasL, leading to immunotherapeutic resistance in obese mice. By increasing intracellular IFN-γ and reducing PD-1 expression, blocking the IL-4 pathway promotes CD8+ T-cell cytotoxicity, thus sensitizing BCCs to anti-cancer therapy and enhancing the immune responses^107^. Another study indicated that the expression of leptin, CXCR4, and CCR9 in obese TNBC patients is negatively correlated with CD8+ T-cell infiltration^95^. Furthermore, ADSCs suppress B-cell proliferation, reduce the TNF-α+:IL-10+ B-cell ratio in a contact-dependent manner, and alter the cytokine profile of B cells to an anti-inflammatory profile^136^. IL-6 inhibition reverses the recruitment of immunosuppressive regulatory T cells induced by anti-VEGF treatment in obese mice, suggesting that IL-6 suppression may augment immunotherapy in the context of obesity^85^. Additionally, pro-tumoral IL-17+ γδT cells multiply in obese mouse tumors, exhibit high lipid absorption, and intracellular lipid storage, indicating that metabolic programming regulates the immune cell lineage and function^137^. Moreover, using 4T1 tumor models in the presence or absence of adipocytes, Liu et al.^91^ noted that TAA-secreted CCL2 recruits monocytes and macrophages to become immunosuppressive MDSCs and M2 macrophages. Targeting CCL2 *in vivo* increases T-cell infiltration, ameliorates the immunosuppressive TME, and facilitates immunotherapy.

Monocytes and macrophages are involved in innate immunity and have an important role in antigen presentation and immune regulation; however, an increasing number of studies have shown that myeloid cells have a large number of immunosuppressive phenotypes that have an important role in mediating tumor immune escape. Obesity-related M1 macrophages secrete IL-6 via a JAK/STAT-dependent pathway to promote PD-L1 expression in TNBC, and telmisartan reverses this process by activating peroxisome proliferator-activated receptor (PPAR-γ) and inhibiting NF-κB P65, thus highlighting the potential application in adjuvant TNBC immunotherapy^138^. Moreover, AnaR-secreted leptin enhances macrophage motility and induces an M2-like phenotype via CXCR4 signaling, highlighting the clinical advantage of targeting the cytokine network in obesity-associated hormone-resistant breast tumors^117^. Consistent with this finding, loss of ObR reduces macrophage recruitment, resulting in decreased CCL2 in xenograft tumors and co-culture experiments. In addition, the absence of Ob/ObR signaling regulates the immunosuppressive TME, as shown by reduced expression of PD-L1/PD-1/arginase 1 and increased phagocytosis capability in macrophages^116^. Accordingly, obesity-associated inflammatory signals promote the formation of an immunosuppressive microenvironment and tumor immune escape by inducing macrophage differentiation into a pro-tumor phenotype and increasing immune checkpoint expression.

## Discussion and future perspectives

**Figure 3:**
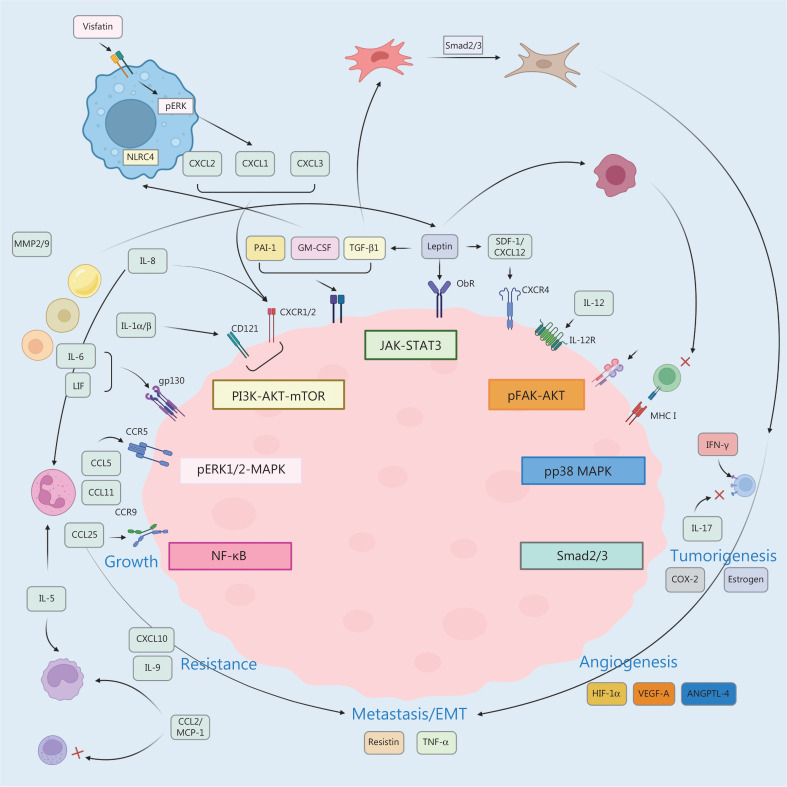
Summarized potential inflammatory pathways that may be involved in obesity-associated TIME. Continuous cell proliferation and differentiation in a microenvironment containing inflammatory cells, growth factors, activated matrices, and DNA-damaged materials are crucial for inflammation to encourage cancer formation. The TIME is the essential condition for differentiation, survival, and metastasis, and inflammatory cells are closely involved in regulating the TIME. The remodeled breast TIME secretes inflammatory mediators that crosstalk with cells by acting on surface receptors, activating inflammation-related signaling pathways, and modifying the behavior of tumor, immune, and stromal cells. In an inflammatory microenvironment, there are three primary stages for tumor formation and development (initiation, promotion, and metastasis). The inflammatory microenvironment involves immune cells, inflammatory cells, and various produced bioactive chemicals, such as TNF-α, IFN-γ, interleukin, and chemokines, which participate in cancer cell tumorigenesis, growth, angiogenesis, metastasis, and resistance to therapeutic approaches.

Despite the well-documented association between obesity and cancer, the underlying mechanisms are unclear. Recognition of obesity as a chronic inflammatory condition, however, implies potential roles for inflammatory cytokines in obesity-associated BC (**Figure 3**). Nevertheless, there remain many unanswered questions.

To determine the disease extent and develop successful treatments, it is essential to recognize cancer as a complex and interconnected process. The inflammatory and obese microenvironment of BC is not confined to the effects of the adipocyte secretome. Stromal cells in the microenvironment release inflammatory mediators to affect cancer behavior and survival. Similarly, cancer cells also induce changes in surrounding cells via the secretome^139^. Interactions among immune cells, cancer cells, adipocytes, the immunome, and the metabolome generate intriguing dynamics and lead to the secretion of important molecules that are likely to be potential subjects for inflammation-targeted therapies in the future (**Table 1**). Although many studies have identified cells and molecules that may have roles in mediating BC, there has been a lack of continuous observations and cell tracking to distinguish different cell types, determine the specific source of inflammatory molecules, or determine whether other microenvironmental factors are involved. Due to the many molecular BC subtypes, diverse interactions and heterogeneity are displayed^72^, yet this finding seldom receives any notice from researchers. Moreover, different adipocyte subpopulations between pre- and post-menopausal breast tissues likely utilize distinct mechanisms to make neighboring epithelial cells sense activated inflammatory signaling and exhibit diverse responses^44^. In brief, variables that may influence the heterogeneity of the TME warrant consideration.

**Table 1:** Potential inflammatory targets of BC

	Agent	Inflammatory pathway	Impact on tumor	Reference
Metastasis				
CXCR4	AMD3100	SDF-1/CXCR4	Bone metastasis	^94^
LIF	EC330	LIF-CXCL1/2/3/8-CXCR2	Migration/invasion; lung metastasis	^97^
CXCR2	SB225002			
CXCR1/2	Reparixin	IL8-CXCR1/2	Migration/invasion	^97,85^
S1P	FTY720/fingolimod	SphK1/S1P/S1PR1	Cytokines/macrophage infiltration	^100^
GLUT	Metformin	GM-CSF/MMP9	Metastasis progression	^87^
STAT3	Stattic	IL6-STAT3	EMT/migration/invasion	^112^
Resistance to Therapy				
Autotaxin	GLPG1690	ATX-LPA	Resistance to radiotherapy	^131^
CCL2	CCL2-binding trap	CCL2-T cell	T-cell infiltration	^91^
Angiotensin receptor II	Telmisartan	PPARγ/NF-κB P65-IL6	PD-L1 expression	^138^
Angiogenesis				
VEGF	B20-4.1.1	VEGF	Angiogenesis	^85^
IL-6	/	IL6-Treg	Reverse anti-VEGF-induced Treg recruitment	
ANGPTL4	/	ANGPTL4	Angiogenesis	^87^
CXCL1/2	SCH527123	CXCL1/8-CXCR1/2	Angiogenesis	^72^
Tumorigenesis/Stemness				
pERK	PD98059	Visfatin-pERK-CXCL1	EMT/migration/invasion; stemness	^51^
COX	Aspirin	Inflammatory/angiogenic mediators	Tumorigenesis	^86^

Tumors grow in a complex microenvironment containing diverse cell types and inflammatory mediators. Beyond the local TME, a systemic inflammatory environment can also influence the disease course by interfering with homeostasis in a variety of tissues across the body. Many studies, however, do not consider *in vivo* inflammation, instead identifying interactions between tumor cells and adipocytes using co-cultivation methods. This approach may overlook the systemic effects of metabolism and immunity and lead to different consequences. Therefore, the complex interplay of inflammatory mediators in the context of an immunocompetent host remains to be explored. Additionally, it is unknown whether any of the current findings can be applied to the microenvironment of human BC; further exploration is needed in more reliable models, such as xenograft mice and organoids.

As a systemic disease, complex interactions occur between obesity and the endocrine and metabolic systems of the body, and the comprehensive effect on the tumor immune microenvironment (TIME) needs to be further explored. Obesity causes altered glucose metabolism and increases circulating FFAs, which results in hyperinsulinemia and insulin resistance, elevates the secretion of pro-inflammatory cytokines, causes excessive activation of insulin receptors, and promotes tumor proliferation. Insulin resistance often occurs along with obesity, and like obesity, insulin resistance influences the TIME through inflammatory pathways, such as IKK-β or NLRP3^140,141^. In addition, Wang et al.^142^ suggested that insulin resistance in vascular endothelial cells forms a pro-inflammatory state, showing higher levels of cell adhesion proteins and neutrophil infiltration, promoting tumor progression. The study suggested that insulin resistance in the TIME is equally heterogeneous; however, additional studies are needed to verify the findings and the roles obesity and insulin resistance have in the inflammatory TIME.

BC exhibits high genotypic and phenotypic diversity, and heterogeneous cell types within the TME exhibit dynamic and tumor-promoting behaviors during cancer progression. Therefore, identifying interactions between individual cells and cytokines, as well as microenvironmental regulators of tumor progression, is essential to develop predictive molecular signatures and more effective treatments. Sophisticated multiomics technologies, such as genomics, proteomics, metabolomics, single-cell omics, and spatial transcriptomics, comprehensively analyze and reflect tumor heterogeneity at the single-cell level. High-throughput data can be used to analyze the spatial structure of the TIME at the level of single-cell clustering, with far-reaching significance for the realization of precise cancer therapy.

Accordingly, for an integrated understanding of cancer, research into how the systemic environment affects tumor biology is essential. Accurate and efficient analysis of the complex TME and evaluating the meaning of biological targets or biomarkers for clinical applications has been challenging for researchers. Predictive models are data-driven algorithms that combine multiple important outcome-related predictors to assess the prognosis, curative efficacy, and risks of developing a disease based on a variety of mathematical modeling approaches. For example, an integrated analysis of multiple databases from Zhao et al.^143^ identified 10 hub genes associated with the extent of immune cell infiltration into the TIME and patient prognosis. The genes may serve as a risk and survival predictor of BC TIME. By applying mathematical methods to clinical medicine, increasingly accurate predictive clinical models have been developed to aid patients and clinicians in making decisions.

With the continuous advances in technology, next-generation sequencing, including whole exon and RNA sequencing, has been gradually utilized in clinical practice, thus providing massive data that help us explore biological factors related to the treatment response and prognosis. Moreover, pathologic images of human tumor tissue contain a large amount of information that people cannot fully distinguish and understand. Artificial intelligence can extract complex information from visual data, digitally analyze histologic tumor slices, and infer molecular and genetic changes in tumor tissues to provide a wide range of clinically-relevant applications. For example, researchers recently constructed a simplified mathematical model to predict systemic immune cell connectivity and the dynamics of immune interactions using a high-throughput surface receptor screening method, thus providing a comprehensive view of the human immune system at the single-cell level that may provide opportunities for clinical therapeutic interventions^144^. High-throughput mathematical and digital models perform well in elucidating biological processes and identifying key factors of clinical significance, which is a subject that needs future investigation and has profound clinical translational value.

## Conclusions

During cancer development, different types of stromal cells establish essential information exchanges with luminal and basal epithelial cells present in the mammary ducts through paracrine signaling or cell-cell communication within the mammary adipose microenvironment. A thorough understanding of this epithelial-stromal interaction might uncover new pathways and mechanisms involved in tumor progression, with an increasing number of studies shifting the targets from the internal communication between cancer cells to the interaction between malignancies and the surroundings. Given the complicated function of the aforementioned obesity-related inflammatory mediators in the TME, addressing the paracrine metabolic reprogramming and immunomodulatory pathways might be an efficient strategy to combat BC. On the premise of clarifying the heterogeneity of the TME, combining cell therapies inhibiting targeted inflammatory molecules with other conventional chemotherapy, immunotherapy, and radiotherapy strategies is a promising direction for treating BC.

## References

[1] Siegel RL, Miller KD, Fuchs HE, Jemal A (2022). Cancer statistics, 2022. CA Cancer J Clin.

[2] Lei S, Zheng R, Zhang S, Chen R, Wang S, Sun K (2021). Breast cancer incidence and mortality in women in China: temporal trends and projections to 2030. Cancer Biol Med.

[3] Heer E, Harper A, Escandor N, Sung H, McCormack V, Fidler-Benaoudia MM (2020). Global burden and trends in premenopausal and postmenopausal breast cancer: a population-based study. Lancet Glob Health.

[4] Matafome P, Santos-Silva D, Sena CM, Seiça R (2013). Common mechanisms of dysfunctional adipose tissue and obesity-related cancers. Diabetes Metab Res Rev.

[5] Place AE, Jin Huh S, Polyak K (2011). The microenvironment in breast cancer progression: biology and implications for treatment. Breast Cancer Res.

[6] Korkaya H, Liu S, Wicha MS (2011). Breast cancer stem cells, cytokine networks, and the tumor microenvironment. J Clin Invest.

[7] Bissell MJ, Hines WC (2011). Why don’t we get more cancer? A proposed role of the microenvironment in restraining cancer progression. Nat Med.

[8] Quail DF, Joyce JA (2013). Microenvironmental regulation of tumor progression and metastasis. Nat Med.

[9] Liu Y, Guo J, Huang L (2020). Modulation of tumor microenvironment for immunotherapy: focus on nanomaterial-based strategies. Theranostics.

[10] Widschwendter P, Friedl TW, Schwentner L, DeGregorio N, Jaeger B, Schramm A (2015). The influence of obesity on survival in early, high-risk breast cancer: results from the randomized SUCCESS A trial. Breast Cancer Res.

[11] Pfeiler G, Stöger H, Dubsky P, Mlineritsch B, Singer C, Balic M (2013). Efficacy of tamoxifen ± aminoglutethimide in normal weight and overweight postmenopausal patients with hormone receptor-positive breast cancer: an analysis of 1509 patients of the ABCSG-06 trial. Br J Cancer.

[12] Biganzoli E, Desmedt C, Fornili M, de Azambuja E, Cornez N, Ries F (2017). Recurrence dynamics of breast cancer according to baseline body mass index. Eur J Cancer.

[13] Copson ER, Cutress RI, Maishman T, Eccles BK, Gerty S, Stanton L (2015). Obesity and the outcome of young breast cancer patients in the UK: the POSH study. Ann Oncol.

[14] Pfeiler G, Königsberg R, Fesl C, Mlineritsch B, Stoeger H, Singer CF (2011). Impact of body mass index on the efficacy of endocrine therapy in premenopausal patients with breast cancer: an analysis of the prospective ABCSG-12 trial. J Clin Oncol.

[15] Crozier JA, Moreno-Aspitia A, Ballman KV, Dueck AC, Pockaj BA, Perez EA (2013). Effect of body mass index on tumor characteristics and disease-free survival in patients from the HER2-positive adjuvant trastuzumab trial N9831. Cancer.

[16] Chen S, Chen CM, Zhou Y, Zhou RJ, Yu KD, Shao ZM (2012). Obesity or overweight is associated with worse pathological response to neoadjuvant chemotherapy among Chinese women with breast cancer. PLoS One.

[17] Pajares B, Pollán M, Martín M, Mackey JR, Lluch A, Gavila J (2013). Obesity and survival in operable breast cancer patients treated with adjuvant anthracyclines and taxanes according to pathological subtypes: a pooled analysis. Breast Cancer Res.

[18] de Azambuja E, McCaskill-Stevens W, Francis P, Quinaux E, Crown JP, Vicente M (2010). The effect of body mass index on overall and disease-free survival in node-positive breast cancer patients treated with docetaxel and doxorubicin-containing adjuvant chemotherapy: the experience of the BIG 02-98 trial. Breast Cancer Res Treat.

[19] Wang H, Zhang S, Yee D, Basu S, Beckwith H, Potter D (2021). Impact of body mass index on pathological complete response following neoadjuvant chemotherapy in operable breast cancer: a meta-analysis. Breast Cancer.

[20] Floris G, Richard F, Hamy AS, Jongen L, Wildiers H, Ardui J (2021). Body mass index and tumor-infiltrating lymphocytes in triple-negative breast cancer. J Natl Cancer Inst.

[21] Lauby-Secretan B, Scoccianti C, Loomis D, Grosse Y, Bianchini F, Straif K (2016). Body fatness and cancer – viewpoint of the IARC Working Group. New Engl J Med.

[22] Deng T, Lyon CJ, Bergin S, Caligiuri MA, Hsueh WA (2016). Obesity, inflammation, and cancer. Annu Rev Pathol.

[23] Demark-Wahnefried W, Rogers LQ, Gibson JT, Harada S, Frugé AD, Oster RA (2020). Randomized trial of weight loss in primary breast cancer: impact on body composition, circulating biomarkers and tumor characteristics. Int J Cancer.

[24] Saha S, Mukherjee S, Khan P, Kajal K, Mazumdar M, Manna A (2016). Aspirin suppresses the acquisition of chemoresistance in breast cancer by disrupting an NFκB-IL6 signaling axis responsible for the generation of cancer stem cells. Cancer Res.

[25] Mantovani A, Allavena P, Sica A, Balkwill F (2008). Cancer-related inflammation. Nature.

[26] Hartman ZC, Poage GM, den Hollander P, Tsimelzon A, Hill J, Panupinthu N (2013). Growth of triple-negative breast cancer cells relies upon coordinate autocrine expression of the proinflammatory cytokines IL-6 and IL-8. Cancer Res.

[27] Miran I, Scherer D, Ostyn P, Mazouni C, Drusch F, Bernard M (2020). Adipose tissue properties in tumor-bearing breasts. Front Oncol.

[28] Mavrogonatou E, Pratsinis H, Kletsas D (2020). The role of senescence in cancer development. Semin Cancer Biol.

[29] Andarawewa KL, Motrescu ER, Chenard MP, Gansmuller A, Stoll I, Tomasetto C (2005). Stromelysin-3 is a potent negative regulator of adipogenesis participating to cancer cell-adipocyte interaction/crosstalk at the tumor invasive front. Cancer Res.

[30] Cozzo AJ, Fuller AM, Makowski L (2017). Contribution of adipose tissue to development of cancer. Compr Physiol.

[31] Choi J, Cha YJ, Koo JS (2018). Adipocyte biology in breast cancer: from silent bystander to active facilitator. Prog Lipid Res.

[32] Zwick RK, Guerrero-Juarez CF, Horsley V, Plikus MV (2018). Anatomical, physiological, and functional diversity of adipose tissue. Cell Metab.

[33] Galic S, Oakhill JS, Steinberg GR (2010). Adipose tissue as an endocrine organ. Mol Cell Endocrinol.

[34] Poulos SP, Hausman DB, Hausman GJ (2010). The development and endocrine functions of adipose tissue. Mol Cell Endocrinol.

[35] Dietze EC, Chavez TA, Seewaldt VL (2018). Obesity and triple-negative breast cancer: disparities, controversies, and biology. Am J Pathol.

[36] Chu DT, Phuong TNT, Tien NLB, Tran DK, Nguyen TT, Thanh VV (2019). The effects of adipocytes on the regulation of breast cancer in the tumor microenvironment: an update. Cells.

[37] Walter M, Liang S, Ghosh S, Hornsby PJ, Li R (2009). Interleukin 6 secreted from adipose stromal cells promotes migration and invasion of breast cancer cells. Oncogene.

[38] Martínez-Chacón G, Brown KA, Docanto MM, Kumar H, Salminen S, Saarinen N (2018). IL-10 suppresses TNF-α-induced expression of human aromatase gene in mammary adipose tissue. FASEB J.

[39] Dirat B, Bochet L, Dabek M, Daviaud D, Dauvillier S, Majed B (2011). Cancer-associated adipocytes exhibit an activated phenotype and contribute to breast cancer invasion. Cancer Res.

[40] Reeves GK, Pirie K, Beral V, Green J, Spencer E, Bull D (2007). Cancer incidence and mortality in relation to body mass index in the Million Women Study: cohort study. BMJ.

[41] Neuhouser ML, Aragaki AK, Prentice RL, Manson JE, Chlebowski R, Carty CL (2015). Overweight, obesity, and postmenopausal invasive breast cancer risk: a secondary analysis of the women’s health initiative randomized clinical trials. JAMA Oncol.

[42] Iyengar NM, Arthur R, Manson JE, Chlebowski RT, Kroenke CH, Peterson L (2019). Association of body fat and risk of breast cancer in postmenopausal women with normal body mass index: a secondary analysis of a Randomized Clinical Trial and Observational Study. JAMA Oncol.

[43] Cecchini RS, Costantino JP, Cauley JA, Cronin WM, Wickerham DL, Land SR (2012). Body mass index and the risk for developing invasive breast cancer among high-risk women in NSABP P-1 and STAR breast cancer prevention trials. Cancer Prev Res (Phila).

[44] Alhallak I, Wolter KG, Castro Munoz A, Simmen FA, Ward RJ, Petty SA (2021). Breast adipose regulation of premenopausal breast epithelial phenotype involves interleukin 10. J Mol Endocrinol.

[45] Martínez-Chacón G, Yatkin E, Polari L, Deniz Dinç D, Peuhu E, Hartiala P (2021). CC chemokine ligand 2 (CCL2) stimulates aromatase gene expression in mammary adipose tissue. FASEB J.

[46] Gonçalves RM, Delgobo M, Agnes JP, das Neves RN, Falchetti M, Casagrande T (2021). COX-2 promotes mammary adipose tissue inflammation, local estrogen biosynthesis, and carcinogenesis in high-sugar/fat diet treated mice. Cancer Lett.

[47] Gonzalez Suarez N, Fernandez-Marrero Y, Torabidastgerdooei S, Annabi B (2022). EGCG prevents the onset of an inflammatory and cancer-associated adipocyte-like phenotype in adipose-derived mesenchymal stem/stromal cells in response to the triple-negative breast cancer secretome. Nutrients.

[48] Roubert A, Gregory K, Li Y, Pfalzer AC, Li J, Schneider SS (2017). The influence of tumor necrosis factor-α on the tumorigenic Wnt-signaling pathway in human mammary tissue from obese women. Oncotarget.

[49] Chamberlin T, Thompson V, Hillers-Ziemer LE, Walton BN, Arendt LM (2020). Obesity reduces mammary epithelial cell TGFβ1 activity through macrophage-mediated extracellular matrix remodeling. FASEB J.

[50] Ginestier C, Wicha MS (2007). Mammary stem cell number as a determinate of breast cancer risk. Breast Cancer Res.

[51] Wang YY, Chen HD, Lo S, Chen YK, Huang YC, Hu SC (2020). Visfatin enhances breast cancer progression through CXCL1 induction in tumor-associated macrophages. Cancers (Basel).

[52] Picon-Ruiz M, Pan C, Drews-Elger K, Jang K, Besser AH, Zhao D (2016). Interactions between adipocytes and breast cancer cells stimulate cytokine production and drive Src/Sox2/miR-302b-mediated malignant progression. Cancer Res.

[53] Tiwari P, Blank A, Cui C, Schoenfelt KQ, Zhou G, Xu Y (2019). Metabolically activated adipose tissue macrophages link obesity to triple-negative breast cancer. J Exp Med.

[54] Chen Y, He Y, Wang X, Lu F, Gao J (2019). Adipose-derived mesenchymal stem cells exhibit tumor tropism and promote tumorsphere formation of breast cancer cells. Oncol Rep.

[55] Avtanski D, Garcia A, Caraballo B, Thangeswaran P, Marin S, Bianco J (2019). Resistin induces breast cancer cells epithelial to mesenchymal transition (EMT) and stemness through both adenylyl cyclase-associated protein 1 (CAP1)-dependent and CAP1-independent mechanisms. Cytokine.

[56] Goto H, Shimono Y, Funakoshi Y, Imamura Y, Toyoda M, Kiyota N (2019). Adipose-derived stem cells enhance human breast cancer growth and cancer stem cell-like properties through adipsin. Oncogene.

[57] Hao J, Zhang Y, Yan X, Yan F, Sun Y, Zeng J (2018). Circulating adipose fatty acid binding protein is a new link underlying obesity-associated breast/mammary tumor development. Cell Metab.

[58] Mishra AK, Parish CR, Wong ML, Licinio J, Blackburn AC (2017). Leptin signals via TGFB1 to promote metastatic potential and stemness in breast cancer. PLoS One.

[59] Silva C, Andrade N, Guimarães JT, Patrício E, Martel F (2021). The in vitro effect of the diabetes-associated markers insulin, leptin and oxidative stress on cellular characteristics promoting breast cancer progression is GLUT1-dependent. Eur J Pharmacol.

[60] Juárez-Cruz JC, Okoniewski M, Ramírez M, Ortuño-Pineda C, Navarro-Tito N, Castañeda-Saucedo E (2022). Chronic leptin treatment induces epithelial-mesenchymal transition in MCF10A mammary epithelial cells. J Mammary Gland Biol Neoplasia.

[61] Bi TQ, Che XM (2010). Nampt/PBEF/visfatin and cancer. Cancer Biol Ther.

[62] Behrouzfar K, Alaee M, Nourbakhsh M, Gholinejad Z, Golestani A (2017). Extracellular NAMPT/visfatin causes p53 deacetylation via NAD production and SIRT1 activation in breast cancer cells. Cell Biochem Funct.

[63] Gholinejad Z, Kheiripour N, Nourbakhsh M, Ilbeigi D, Behroozfar K, Hesari Z (2017). Extracellular NAMPT/Visfatin induces proliferation through ERK1/2 and AKT and inhibits apoptosis in breast cancer cells. Peptides.

[64] Ka NL, Lim GY, Kim SS, Hwang S, Han J, Lee YH (2022). Type I IFN stimulates IFI16-mediated aromatase expression in adipocytes that promotes E(2)-dependent growth of ER-positive breast cancer. Cell Mol Life Sci.

[65] Kuziel G, Thompson V, D’Amato JV, Arendt LM (2020). Stromal CCL2 signaling promotes mammary tumor fibrosis through recruitment of myeloid-lineage cells. Cancers (Basel).

[66] Kim HS, Jung M, Choi SK, Woo J, Piao YJ, Hwang EH (2018). IL-6-mediated cross-talk between human preadipocytes and ductal carcinoma in situ in breast cancer progression. J Exp Clin Cancer Res.

[67] Jimenez T, Friedman T, Vadgama J, Singh V, Tucker A, Collazo J (2020). Nicotine synergizes with high-fat diet to induce an anti-inflammatory microenvironment to promote breast tumor growth. Mediators Inflamm.

[68] Fletcher SJ, Hapon MB, Callegari EA, Crosbie ML, Santiso N, Ursino A (2018). Comparative proteomics of soluble factors secreted by human breast adipose tissue from tumor and normal breast. Oncotarget.

[69] Lee J, Hong BS, Ryu HS, Lee HB, Lee M, Park IA (2017). Transition into inflammatory cancer-associated adipocytes in breast cancer microenvironment requires microRNA regulatory mechanism. PLoS One.

[70] Lyes MA, Payne S, Ferrell P, Pizzo SV, Hollenbeck ST, Bachelder RE (2019). Adipose stem cell crosstalk with chemo-residual breast cancer cells: implications for tumor recurrence. Breast Cancer Res Treat.

[71] Zhao Y, Zhang X, Zhao H, Wang J, Zhang Q (2018). CXCL5 secreted from adipose tissue-derived stem cells promotes cancer cell proliferation. Oncol Lett.

[72] Wang Y, Liu J, Jiang Q, Deng J, Xu F, Chen X (2017). Human adipose-derived mesenchymal stem cell-secreted CXCL1 and CXCL8 facilitate breast tumor growth by promoting angiogenesis. Stem Cells.

[73] Li W, Xu H, Qian C (2017). c-Kit-positive adipose tissue-derived mesenchymal stem cells promote the growth and angiogenesis of breast cancer. Biomed Res Int.

[74] Rivera LB, Bergers G (2015). CANCER. Tumor angiogenesis, from foe to friend. Science.

[75] Jain RK (2013). Normalizing tumor microenvironment to treat cancer: bench to bedside to biomarkers. J Clin Oncol.

[76] Carmeliet P, Jain RK (2011). Molecular mechanisms and clinical applications of angiogenesis. Nature.

[77] Carmeliet P, Jain RK (2011). Principles and mechanisms of vessel normalization for cancer and other angiogenic diseases. Nat Rev Drug Discov.

[78] Costa C, Incio J, Soares R (2007). Angiogenesis and chronic inflammation: cause or consequence?. Angiogenesis.

[79] Hosogai N, Fukuhara A, Oshima K, Miyata Y, Tanaka S, Segawa K (2007). Adipose tissue hypoxia in obesity and its impact on adipocytokine dysregulation. Diabetes.

[80] Lijnen HR (2008). Angiogenesis and obesity. Cardiovasc Res.

[81] Gu JW, Young E, Patterson SG, Makey KL, Wells J, Huang M (2011). Postmenopausal obesity promotes tumor angiogenesis and breast cancer progression in mice. Cancer Biol Ther.

[82] Arendt LM, McCready J, Keller PJ, Baker DD, Naber SP, Seewaldt V (2013). Obesity promotes breast cancer by CCL2-mediated macrophage recruitment and angiogenesis. Cancer Res.

[83] Limaverde-Sousa G, Sternberg C, Ferreira CG (2014). Antiangiogenesis beyond VEGF inhibition: a journey from antiangiogenic single-target to broad-spectrum agents. Cancer Treat Rev.

[84] Vazquez Rodriguez G, Abrahamsson A, Jensen LDE, Dabrosin C (2018). Adipocytes promote early steps of breast cancer cell dissemination via interleukin-8. Front Immunol.

[85] Incio J, Ligibel JA, McManus DT, Suboj P, Jung K, Kawaguchi K (2018). Obesity promotes resistance to anti-VEGF therapy in breast cancer by up-regulating IL-6 and potentially FGF-2. Sci Transl Med.

[86] Hsieh CC, Chiu HH, Wang CH, Kuo CH (2020). Aspirin modifies inflammatory mediators and metabolomic profiles and contributes to the suppression of obesity-associated breast cancer cell growth. Int J Mol Sci.

[87] Kolb R, Kluz P, Tan ZW, Borcherding N, Bormann N, Vishwakarma A (2019). Obesity-associated inflammation promotes angiogenesis and breast cancer via angiopoietin-like 4. Oncogene.

[88] Reggiani F, Labanca V, Mancuso P, Rabascio C, Talarico G, Orecchioni S (2017). Adipose progenitor cell secretion of GM-CSF and mmp9 promotes a stromal and immunological microenvironment that supports breast cancer progression. Cancer Res.

[89] Wu M, Chen L, Qi Y, Ci H, Mou S, Yang J (2022). Human umbilical cord mesenchymal stem cell promotes angiogenesis via integrin β1/ERK1/2/HIF-1α/VEGF-A signaling pathway for off-the-shelf breast tissue engineering. Stem Cell Res Ther.

[90] Karin N (2018). Chemokines and cancer: new immune checkpoints for cancer therapy. Curr Opin Immunol.

[91] Liu Y, Tiruthani K, Wang M, Zhou X, Qiu N, Xiong Y (2021). Tumor-targeted gene therapy with lipid nanoparticles inhibits tumor-associated adipocytes and remodels the immunosuppressive tumor microenvironment in triple-negative breast cancer. Nanoscale Horiz.

[92] D’Esposito V, Liguoro D, Ambrosio MR, Collina F, Cantile M, Spinelli R (2016). Adipose microenvironment promotes triple negative breast cancer cell invasiveness and dissemination by producing CCL5. Oncotarget.

[93] Zhong X, Wang X, Sun Q (2021). CCL2/ACKR2 interaction participate in breast cancer metastasis especially in patients with altered lipid metabolism. Med Hypotheses.

[94] Duan L, Lu Y, Xie W, Nong L, Jia Y, Tan A (2020). Leptin promotes bone metastasis of breast cancer by activating the SDF-1/CXCR4 axis. Aging (Albany NY).

[95] Evangelista GCM, Salvador PA, Soares SMA, Barros LRC, Xavier F, Abdo LM (2019). 4T1 mammary carcinoma colonization of metastatic niches is accelerated by obesity. Front Oncol.

[96] Wu Q, Li B, Li Z, Li J, Sun S, Sun S (2019). Cancer-associated adipocytes: key players in breast cancer progression. J Hematol Oncol.

[97] Zhou C, He X, Tong C, Li H, Xie C, Wu Y (2022). Cancer-associated adipocytes promote the invasion and metastasis in breast cancer through LIF/CXCLs positive feedback loop. Int J Biol Sci.

[98] Al-Khalaf HH, Al-Harbi B, Al-Sayed A, Arafah M, Tulbah A, Jarman A (2019). Interleukin-8 activates breast cancer-associated adipocytes and promotes their angiogenesis- and tumorigenesis-promoting effects. Mol Cell Biol.

[99] Nickel A, Blücher C, Kadri OA, Schwagarus N, Müller S, Schaab M (2018). Adipocytes induce distinct gene expression profiles in mammary tumor cells and enhance inflammatory signaling in invasive breast cancer cells. Sci Rep.

[100] Nagahashi M, Yamada A, Katsuta E, Aoyagi T, Huang WC, Terracina KP (2018). Targeting the SphK1/S1P/S1PR1 axis that links obesity, chronic inflammation, and breast cancer metastasis. Cancer Res.

[101] Martinez-Bernabe T, Sastre-Serra J, Ciobu N, Oliver J, Pons DG, Roca P (2021). Estrogen receptor beta (ERβ) maintains mitochondrial network regulating invasiveness in an obesity-related inflammation condition in breast cancer. Antioxidants (Basel).

[102] Park JY, Kang SE, Ahn KS, Um JY, Yang WM, Yun M (2020). Inhibition of the PI3K-AKT-mTOR pathway suppresses the adipocyte-mediated proliferation and migration of breast cancer cells. J Cancer.

[103] He JY, Wei XH, Li SJ, Liu Y, Hu HL, Li ZZ (2018). Adipocyte-derived IL-6 and leptin promote breast cancer metastasis via upregulation of lysyl hydroxylase-2 expression. Cell Commun Signal.

[104] Gyamfi J, Lee YH, Eom M, Choi J (2018). Interleukin-6/STAT3 signalling regulates adipocyte induced epithelial-mesenchymal transition in breast cancer cells. Sci Rep.

[105] Doi K, Ogata F, Eto H, Kato H, Kuno S, Kinoshita K (2015). Differential contributions of graft-derived and host-derived cells in tissue regeneration/remodeling after fat grafting. Plast Reconstr Surg.

[106] Kengelbach-Weigand A, Tasbihi K, Strissel PL, Schmid R, Marques JM, Beier JP (2019). Plasticity of patient-matched normal mammary epithelial cells is dependent on autologous adipose-derived stem cells. Sci Rep.

[107] Gaggianesi M, Turdo A, Chinnici A, Lipari E, Apuzzo T, Benfante A (2017). IL4 primes the dynamics of breast cancer progression via DUSP4 inhibition. Cancer Res.

[108] Wu S, Wang Y, Yuan Z, Wang S, Du H, Liu X (2019). Human adipose-derived mesenchymal stem cells promote breast cancer MCF7 cell epithelial-mesenchymal transition by cross interacting with the TGF-β/Smad and PI3K/AKT signaling pathways. Mol Med Rep.

[109] Chang YH, Hoang NN, Khanh VC, Yamashita T, Osaka M, Hiramatsu Y (2022). Type 2 diabetes mellitus promotes the differentiation of adipose tissue-derived mesenchymal stem cells into cancer-associated fibroblasts, induced by breast cancer cells. Stem Cells Dev.

[110] Hillers-Ziemer LE, Williams AE, Janquart A, Grogan C, Thompson V, Sanchez A (2021). Obesity-activated lung stromal cells promote myeloid lineage cell accumulation and breast cancer metastasis. Cancers (Basel).

[111] Hiraga T, Ito S, Mizoguchi T (2021). Opposing effects of granulocyte colony-stimulating factor on the initiation and progression of breast cancer bone metastases. Mol Cancer Res.

[112] Liu L, Wu Y, Zhang C, Zhou C, Li Y, Zeng Y (2020). Cancer-associated adipocyte-derived G-CSF promotes breast cancer malignancy via Stat3 signaling. J Mol Cell Biol.

[113] Wu Y, Yu X, Yi X, Wu K, Dwabe S, Atefi M (2017). Aberrant phosphorylation of SMAD4 Thr277-mediated USP9x-SMAD4 interaction by free fatty acids promotes breast cancer metastasis. Cancer Res.

[114] Wolfson B, Zhang Y, Gernapudi R, Duru N, Yao Y, Lo PK (2017). A high-fat diet promotes mammary gland myofibroblast differentiation through microRNA 140 downregulation. Mol Cell Biol.

[115] Quail DF, Olson OC, Bhardwaj P, Walsh LA, Akkari L, Quick ML (2017). Obesity alters the lung myeloid cell landscape to enhance breast cancer metastasis through IL5 and GM-CSF. Nat Cell Biol.

[116] Gelsomino L, Naimo GD, Malivindi R, Augimeri G, Panza S, Giordano C (2020). Knockdown of leptin receptor affects macrophage phenotype in the tumor microenvironment inhibiting breast cancer growth and progression. Cancers (Basel).

[117] Gelsomino L, Giordano C, Camera G, Sisci D, Marsico S, Campana A (2020). Leptin signaling contributes to aromatase inhibitor resistant breast cancer cell growth and activation of macrophages. Biomolecules.

[118] Clements VK, Long T, Long R, Figley C, Smith DMC, Ostrand-Rosenberg S (2018). Frontline science: high fat diet and leptin promote tumor progression by inducing myeloid-derived suppressor cells. J Leukocyte Biol.

[119] Hao J, Yan F, Zhang Y, Triplett A, Zhang Y, Schultz DA (2018). Expression of adipocyte/macrophage fatty acid-binding protein in tumor-associated macrophages promotes breast cancer progression. Cancer Res.

[120] Meads MB, Gatenby RA, Dalton WS (2009). Environment-mediated drug resistance: a major contributor to minimal residual disease. Nat Rev Cancer.

[121] D’Esposito V, Passaretti F, Hammarstedt A, Liguoro D, Terracciano D, Molea G (2012). Adipocyte-released insulin-like growth factor-1 is regulated by glucose and fatty acids and controls breast cancer cell growth in vitro. Diabetologia.

[122] Bochet L, Meulle A, Imbert S, Salles B, Valet P, Muller C (2011). Cancer-associated adipocytes promotes breast tumor radioresistance. Biochem Biophys Res Commun.

[123] Sheng X, Parmentier JH, Tucci J, Pei H, Cortez-Toledo O, Dieli-Conwright CM (2017). Adipocytes sequester and metabolize the chemotherapeutic daunorubicin. Mol Cancer Res.

[124] Bougaret L, Delort L, Billard H, Le Huede C, Boby C, De la Foye A (2018). Adipocyte/breast cancer cell crosstalk in obesity interferes with the anti-proliferative efficacy of tamoxifen. PLoS One.

[125] Yeh WL, Tsai CF, Chen DR (2017). Peri-foci adipose-derived stem cells promote chemoresistance in breast cancer. Stem Cell Res Ther.

[126] Mentoor I, Nell T, Emjedi Z, van Jaarsveld PJ, de Jager L, Engelbrecht AM (2020). Decreased efficacy of doxorubicin corresponds with modifications in lipid metabolism markers and fatty acid profiles in breast tumors from obese vs. lean mice. Front Oncol.

[127] Lumniczky K, Candéias SM, Gaipl US, Frey B (2017). Editorial: radiation and the immune system: current knowledge and future perspectives. Front Immunol.

[128] Balogh A, Shimizu Y, Lee SC, Norman DD, Gangwar R, Bavaria M (2015). The autotaxin-LPA2 GPCR axis is modulated by γ-irradiation and facilitates DNA damage repair. Cell Signal.

[129] Meng G, Tang X, Yang Z, Benesch MGK, Marshall A, Murray D (2017). Implications for breast cancer treatment from increased autotaxin production in adipose tissue after radiotherapy. FASEB J.

[130] Meng G, Wuest M, Tang X, Dufour J, Zhao Y, Curtis JM (2019). Repeated fractions of X-radiation to the breast fat pads of mice augment activation of the autotaxin-lysophosphatidate-inflammatory cycle. Cancers (Basel).

[131] Tang X, Wuest M, Benesch MGK, Dufour J, Zhao Y, Curtis JM (2020). Inhibition of autotaxin with GLPG1690 increases the efficacy of radiotherapy and chemotherapy in a mouse model of breast cancer. Mol Cancer Ther.

[132] Liu Y, Qiu N, Shen L, Liu Q, Zhang J, Cheng YY (2020). Nanocarrier-mediated immunogenic chemotherapy for triple negative breast cancer. J Controlled Release.

[133] Schmid P, Adams S, Rugo HS, Schneeweiss A, Barrios CH, Iwata H (2018). Atezolizumab and nab-paclitaxel in advanced triple-negative breast cancer. New Engl J Med.

[134] Yoo SK, Chowell D, Valero C, Morris LGT, Chan TA (2022). Outcomes among patients with or without obesity and with cancer following treatment with immune checkpoint blockade. JAMA Netw Open.

[135] Gibson JT, Orlandella RM, Turbitt WJ, Behring M, Manne U, Sorge RE (2020). Obesity-associated myeloid-derived suppressor cells promote apoptosis of tumor-infiltrating CD8 T cells and immunotherapy resistance in breast cancer. Front Immunol.

[136] Mehdipour F, Razmkhah M, Rezaeifard S, Bagheri M, Talei AR, Khalatbari B (2018). Mesenchymal stem cells induced anti-inflammatory features in B cells from breast tumor draining lymph nodes. Cell Biol Int.

[137] Lopes N, McIntyre C, Martin S, Raverdeau M, Sumaria N, Kohlgruber AC (2021). Distinct metabolic programs established in the thymus control effector functions of γδ T cell subsets in tumor microenvironments. Nat Immunol.

[138] Wang Y, Zhang X, Xie X, Chen W, Li M, Diao D (2020). Obesity and metabolic syndrome related macrophage promotes PD-L1 expression in TNBC through IL6/JAK/STAT pathway and can be reversed by telmisartan. Cancer Biol Ther.

[139] Liu Y, Wang M, Deng T, Liu R, Ning T, Bai M (2022). Exosomal miR-155 from gastric cancer induces cancer-associated cachexia by suppressing adipogenesis and promoting brown adipose differentiation via C/EPBβ. Cancer Biol Med.

[140] Rose DP, Gracheck PJ, Vona-Davis L (2015). The interactions of obesity, inflammation and insulin resistance in breast cancer. Cancers (Basel).

[141] Iyengar NM, Gucalp A, Dannenberg AJ, Hudis CA (2016). Obesity and cancer mechanisms: tumor microenvironment and inflammation. J Clin Oncol.

[142] Wang X, Häring MF, Rathjen T, Lockhart SM, Sørensen D, Ussar S (2017). Insulin resistance in vascular endothelial cells promotes intestinal tumour formation. Oncogene.

[143] Zhao H, Dang R, Zhu Y, Qu B, Sayyed Y, Wen Y (2022). Hub genes associated with immune cell infiltration in breast cancer, identified through bioinformatic analyses of multiple datasets. Cancer Biol Med.

[144] Shilts J, Severin Y, Galaway F, Müller-Sienerth N, Chong ZS, Pritchard S (2022). A physical wiring diagram for the human immune system. Nature.

